# Understanding coach burnout in high-performance sport: the sequential effects of knowledge, psychological safety, and coach–athlete relationships

**DOI:** 10.3389/fpsyg.2026.1785482

**Published:** 2026-05-08

**Authors:** Rancheng Tao, Ningyi Zhang, Ce Guo

**Affiliations:** School of Athletic Performance, Shanghai University of Sport, Shanghai, China

**Keywords:** coach burnout, coach knowledge, coach–athlete relationship, elite football coaches, psychological safety

## Abstract

**Background:**

Coach burnout is a persistent concern in high-performance sport, yet limited research has examined how upstream cognitive resources contribute to coaches’ wellbeing through psychological and relational processes. Drawing on coaching effectiveness and relational models, this study examined how coach knowledge relates to coach burnout via psychological safety and coach–athlete relationship quality.

**Methods:**

A sample of elite football coaches completed validated measures of coach knowledge (CKQ), sport psychological safety (SPSI), coach–athlete relationship quality (CART-Q), and coach burnout (CBQ). Structural equation modelling (SEM) was used to test a sequential mediation model.

**Results:**

Coach knowledge was positively associated with psychological safety and, both directly and indirectly, with coach–athlete relationship quality. Psychological safety did not independently mediate the association between coach knowledge and burnout. However, a significant sequential indirect effect emerged, indicating that coach knowledge was associated with lower burnout through psychological safety and, subsequently, higher-quality coach–athlete relationships. Coach–athlete relationship quality was directly and negatively associated with burnout.

**Conclusion:**

The findings indicate that coach knowledge becomes consequential for wellbeing primarily through relational pathways, with psychological safety shaping the conditions under which knowledge is enacted within coach–athlete relationships. These results underscore the importance of fostering psychologically safe environments and sustaining high-quality relationships, alongside knowledge development, in efforts to reduce coach burnout in high-performance sport.

## 1 Introduction

Coaching in high-performance sport is widely recognized as a psychologically demanding occupation. Coaches are required not only to deliver technical and tactical expertise but also to manage persistent performance pressures, emotional labor, role ambiguity, and job insecurity (Altfeld et al., 2015; Bentzen et al., 2016). Accumulating evidence suggests that such conditions place coaches at heightened risk of burnout, characterized by emotional and physical exhaustion, sport devaluation, and a reduced sense of accomplishment (Ackeret et al., 2022; Fletcher and Scott, 2010; Lundkvist et al., 2014; Malinauskas et al., 2010; Raedeke and Kenttä, 2013). Although the literature on coach burnout has grown steadily, much of the existing research has focused on stressors, workload, or organizational conditions as primary antecedents. Comparatively less attention has been paid to how positive resources are translated into relational and contextual processes that may protect coaches from burnout. Given the inherently relational nature of coaching, particularly in elite sport settings, examining how coaches’ knowledge resources are associated with the psychological climate they perceive and the quality of their relationships with athletes may offer a more nuanced understanding of coach burnout.

### 1.1 Coach knowledge as a foundational resource

Coach knowledge has been consistently identified as a core component of coaching expertise and effectiveness (Côté and Gilbert, 2009). Contemporary coaching frameworks conceptualize coach knowledge as a multidimensional cognitive resource encompassing professional, interpersonal, and intrapersonal domains (Côté and Gilbert, 2009; Lyle and Cushion, 2016; Jowett, 2017). Such knowledge supports coaches’ capacity to design training, guide athlete development, manage interpersonal interactions, and reflect on their own practice. Rather than representing static technical information, coach knowledge shapes how coaches interpret situational demands, regulate behavior, and respond adaptively to performance-related challenges (Gilbert and Côté, 2013).

Although empirical studies have predominantly linked coach knowledge to coaching effectiveness and performance outcomes, its implications for coaches’ psychological functioning have received limited attention from researchers (Lyle and Cushion, 2016; Quinaud et al., 2022). Nevertheless, theoretical accounts suggest that knowledge resources may influence coaches’ wellbeing indirectly by shaping how they structure training environments, communicate expectations, and engage in reflective regulation under pressure (Gilbert and Côté, 2013; Stodter and Cushion, 2019). Coaches with more developed knowledge bases may draw on their professional expertise and accumulated experience to address situational challenges, which may help to reduce uncertainty and perceived threat in demanding performance contexts (Stodter and Cushion, 2019). In turn, these capacities may influence the broader social and psychological climate in which coaching takes place. In this sense, coach knowledge can be viewed not only as a technical resource, but also as a foundational condition through which psychological and relational processes relevant to burnout may emerge.

### 1.2 Psychological safety as a contextual mechanism

Psychological safety refers to the belief that individuals can take interpersonal risks, such as speaking up, admitting mistakes, or asking for help, without fear of negative consequences (Edmondson, 1999). While this concept originated in organizational research focusing on interpersonal risk-taking within teams, recent sport research has extended psychological safety to include mental-health-related aspects of safety within elite sport environments, reflecting whether individuals perceive the environment as supportive of mental health disclosure, help-seeking, and psychological wellbeing (Henriksen et al., 2020; Rice et al., 2022; Vella et al., 2024). Rice et al. (2022) argue that traditional organizational measures of psychological safety do not adequately capture the specific cultural and stigma-related barriers surrounding mental health disclosure in elite sport contexts. As a result, they developed the Sport Psychological Safety Inventory (SPSI) as a sport-specific measure assessing perceptions of psychological safety related to mental health culture within high-performance sport systems. The SPSI conceptualizes psychological safety through three domains: Mentally Healthy Environment, Mental Health Literacy, and Low Self-Stigma, reflecting organizational support for mental health, knowledge about psychological wellbeing, and reduced stigma surrounding mental health difficulties (Rice et al., 2022).

Within high-performance sport, emerging evidence suggests that psychological safety plays a critical role in linking leadership processes to both performance and wellbeing outcomes. Reviews of organizational research consistently identify leadership as a central antecedent of psychological safety (Edmondson and Lei, 2014), with leaders playing a key role in shaping collective psychological states such as safety and openness (Edmondson and Harvey, 2018). Empirical sport research supports this view. Fransen et al. (2020) demonstrated that psychological safety mediates the relationship between identity leadership and athlete burnout, indicating that psychologically safe environments can buffer the detrimental effects of sustained performance pressure. Similarly, Gosai et al. (2023) found that coaches’ transformational leadership behaviors predict both team psychological safety and coach–athlete relationship quality, with downstream benefits for athletes’ functioning and affective experiences. From the athlete perspective, recent evidence further suggests that psychological safety helps explain the association between athletes’ communication competence, such as openness and effective conflict management, and the quality of the coach–athlete relationship (Jowett et al., 2023). From the coach perspective, higher levels of psychological safety have been shown to predict lower exhaustion and burnout, as well as more positive help-seeking attitudes (Slowiak et al., 2024).

Despite this growing body of research, to our knowledge, no empirical studies have directly examined the relationship between coach knowledge and coaches’ own perceptions of psychological safety. Accordingly, in the present study psychological safety is understood as perceived mental-health-related psychological safety within the elite sport environment, referring to the extent to which coaches perceive their sporting context as supportive of psychological wellbeing, open discussion of mental health concerns, and low stigma around mental health challenges.

### 1.3 Coach–athlete relationship as a relational mechanism

The coach–athlete relationship has long been regarded as the core of the coaching process, reflecting the quality of interpersonal interactions between coaches and athletes in training and competition settings. Conceptually, coach–athlete relationship is commonly defined through the 3 + 1 Cs framework, encompassing affective closeness (emotional bonds and mutual trust), commitment (long-term intention to maintain the relationship), and complementarity (cooperative and responsive interactions), together with co-orientation as the degree of shared understanding between relational partners (Jowett and Ntoumanis, 2004; Jowett and Poczwardowski, 2007).

A substantial body of research has demonstrated that high-quality coach–athlete relationship is associated with a range of adaptive outcomes, including enhanced motivation, greater satisfaction, improved performance, and more positive emotional experiences for both athletes and coaches (Davis et al., 2019; Isoard-Gautheur et al., 2016; Jowett and Poczwardowski, 2007; McShan and Moore, 2023; Thelwell et al., 2017). From the coach’s perspective in particular, recent systematic evidence indicates that the coach–athlete relationship quality is consistently and positively related to coaching satisfaction and perceived effectiveness (McShan and Moore, 2023). These findings highlight the coach–athlete relationship as a critical relational resource within the coaching environment. Importantly, the coach–athlete relationship quality is embedded within a broader psychological and social environment shaped by practices, communication norms, and the emotional climate of the team or organisation (Jowett and Poczwardowski, 2007; Rhind and Jowett, 2012).

### 1.4 Coach burnout as a distal outcome

Coach burnout is typically conceptualized as a multidimensional syndrome comprising emotional and physical exhaustion, sport devaluation, and a reduced sense of accomplishment (Raedeke and Smith, 2001). In high-performance sport, burnout has been recognized as a prevalent and persistent concern, driven by chronic performance pressure, extensive emotional labor, and prolonged exposure to organizational stressors such as job insecurity, role ambiguity, time pressure, and evaluative scrutiny (Bentzen et al., 2016; Fletcher and Scott, 2010; Malinauskas et al., 2010).

Contemporary burnout frameworks emphasize that burnout does not emerge solely from workload or individual vulnerability, but from the sustained interaction between personal resources and the social–organizational context in which work is embedded (Gustafsson et al., 2017; Leiter, 2018; Schaufeli, 2006). In coaching environments, relational demands, particularly the need to manage athletes’ emotions, maintain authority, and sustain motivation under performance pressure, constitute a substantial and ongoing source of emotional labor (Olusoga et al., 2019). Empirical studies in sport settings indicate that higher-quality relationships are associated with lower burnout-related symptoms, suggesting that relational functioning may buffer against emotional exhaustion and sport devaluation (Westfall et al., 2018). However, when these relational demands are experienced as chronically strained or misaligned, they may contribute to the gradual erosion of energy, meaning, and perceived effectiveness that characterizes burnout.

Longitudinal evidence suggests that coach burnout is not merely a transient reaction to situational stressors, but a condition that can accumulate over time when demands persist without adequate psychological or social resources. For example, Ackeret et al. (2022) demonstrated both rank-order and mean-level stability of burnout symptoms among high-performance coaches across a six-month period, while Altfeld et al. (2015) showed that fluctuations in exhaustion and devaluation are shaped by ongoing seasonal stress exposure rather than isolated events. These findings underscore the importance of identifying protective processes that can interrupt the gradual erosion of motivation, engagement, and perceived effectiveness in coaching roles.

### 1.5 The present study

Despite these advances, existing research has predominantly examined coach knowledge in relation to coaching effectiveness and performance outcomes, with limited attention to its implications for coaches’ psychological wellbeing. Little is known about how different forms of coach knowledge contribute to psychological and relational resources that may protect against burnout, or how such resources are enacted within coach–athlete relationships over time. Although constructs such as psychological safety and relationship quality have each been linked to burnout, they are rarely integrated into explanatory models that trace burnout back to cognitive antecedents. As a result, current literature provides limited insight into how cognitive, psychological, and relational processes jointly shape burnout trajectories in elite coaching contexts.

The present study aims to develop and test an integrative structural equation model examining the pathways from coach knowledge to coach burnout via psychological safety and the coach–athlete relationship. By simultaneously considering individual resources, contextual perceptions, and interpersonal processes, this study seeks to advance understanding of the coach burnout in high-performance sport and to inform psychologically grounded coach support and intervention strategies. Based on the above theoretical framework and empirical evidence, the following hypotheses are proposed:

Hypothesis 1: Coach knowledge will be positively associated with psychological safety.

Hypothesis 2: Psychological safety will mediate the relationship between coach knowledge and coach–athlete relationship quality.

Hypothesis 3: Coach–athlete relationship quality will mediate the relationship between coach knowledge and coach burnout.

Hypothesis 4: Coach knowledge will be indirectly associated with coach burnout through a sequential mediation pathway involving psychological safety and coach–athlete relationship quality.

## 2 Method

### 2.1 Participants and procedure

A cross-sectional survey design was employed. Participants were recruited through professional coaching networks and football organizations using purposive sampling. Eligibility criteria included: (a) hold an Asian Football Confederation (AFC) B-level coaching licence or higher, (b) currently serving as a coach at the time of data collection, and (c) have direct responsibility for training design, implementation, and athlete management. A total of 221 coaches were approached.

The study sample consisted primarily of male coaches (*n* = 193, 87.33%), while female coaches accounted for 12.67% (*n* = 28) of participants. Most coaches were concentrated in mid-career stages, with 43.44% aged 31–40 years and 37.10% aged 41–50 years. Participants were engaged across multiple levels of the football performance system. The largest proportion worked in university settings (30.32%), followed by high-level youth training institutions (19.46%). Coaches from professional club first teams (15.84%) and professional reserve teams (13.57%) were also well represented, alongside those coaching high school teams (10.86%), provincial teams (7.69%), and national teams (2.26%). The majority of coaches held upper-tier qualifications, with A-level licenses (52.94%) being most common, followed by B-level licenses (36.65%); a smaller proportion (10.41%) reported holding professional-level certifications. In addition, nearly half of the participants (48.87%) had remained with their current team for more than 6 years.

Data were collected using an anonymous online questionnaire administered via Wenjuanxing, an online survey platform in China that is commonly employed in academic research and provides standardized procedures for data security and participant anonymity. Ethical approval was obtained from the Ethics Committee of Shanghai University of Sport prior to data collection. Potential participants were approached through on-site visits and WeChat invitations to assess their willingness to participate. Coaches who agreed to take part received a participant information sheet describing the purpose of the study, study procedures, and ethical considerations, together with an informed consent form. Upon providing informed consent, participants were invited to complete a multi-section online questionnaire. Participants were informed of the confidentiality and anonymity of their responses, the voluntary nature of participation, and their right to withdraw from the study at any time without penalty (Podsakoff et al., 2003).

### 2.2 Measures

All questionnaires were translated into Mandarin Chinese using a collaborative and iterative translation procedure combining back-translation with conceptual review, in line with recommendations by Douglas and Craig (2007). The original English instruments were translated into Chinese by the first author, who has advanced academic proficiency in both languages, and independently back-translated into English by the second author. Discrepancies were resolved through joint discussion, with emphasis on conceptual equivalence, response scale interpretation, and contextual relevance within elite sport and coaching settings.

Coach knowledge was assessed using the Coach Knowledge Questionnaire (CKQ) developed and validated by Quinaud et al. (2022). The CKQ comprises 13 items, loading on two empirically supported dimensions: PIK (8 items) and IK (5 items), rated on a five-point Likert scale ranging from 1 (strongly disagree) to 5 (strongly agree). Previous validation studies have reported strong reliability for both dimensions, with intra-class correlation coefficients of 0.89 for PIK and 0.83 for IK (Quinaud et al., 2022). In the present sample, the CKQ demonstrated excellent internal consistency (Cronbach’s *α* = 0.93).

Psychological safety was assessed using the Sport Psychological Safety Inventory (SPSI), a sport-specific measure developed and psychometrically validated for use in elite sport settings (Rice et al., 2022). The SPSI comprises three dimensions reflecting key components of psychological safety in sport settings: Mentally Healthy Environment, Mental Health Literacy, and Low Self-Stigma, reflecting perceptions of organizational support for mental health, knowledge and understanding of psychological symptoms, and reduced stigma around mental health challenges within sport systems. Items were rated on a five-point Likert scale ranging from 1 (strongly disagree) to 5 (strongly agree), with higher scores indicating greater perceived psychological safety. Consistent with the original validation study, the negatively worded items were reverse coded prior to analysis. Previous psychometric evaluation has demonstrated acceptable internal consistency for the overall SPSI (Cronbach’s *α* = 0.71) and supported its construct validity in samples of elite athletes, coaches, and high-performance support staff (Rice et al., 2022).

Coach–athlete relationship quality was assessed using the Coach–Athlete Relationship Questionnaire (CART-Q) (Jowett and Ntoumanis, 2004), adapted for the coach self-report perspective. The CART-Q consists of 11 items measuring three relational dimensions: closeness (4 items), commitment (3 items), and complementarity (4 items). Responses were recorded on a seven-point Likert scale ranging from 1 (strongly disagree) to 7 (strongly agree). Previous research has demonstrated sound psychometric properties of the CART-Q across cultural and sport contexts (Jowett and Ntoumanis, 2004; Yang and Jowett, 2013). In the present sample, the CART-Q demonstrated excellent internal consistency (Cronbach’s *α* = 0.91).

Coach burnout was assessed using the Coach Burnout Questionnaire (CBQ; Malinauskas et al., 2010). The CBQ consists of 15 items assessing three dimensions: emotional exhaustion, sport devaluation, and reduced sense of accomplishment, with five items per dimension. Items were rated on a five-point Likert scale (1 = strongly disagree, 5 = strongly agree), with higher scores indicating greater burnout. Within the reduced sense of accomplishment subscale, two positively worded items (Items 1 and 4) were reverse coded prior to analysis so that higher scores consistently reflected higher levels of burnout across all dimensions. Consistent with previous research, the CBQ demonstrated good internal consistency in the present study (Cronbach’s *α* = 0.88).

### 2.3 Data preparation and preliminary checks

Prior to hypothesis testing, the dataset was examined for data quality and underlying assumptions relevant to covariance-based structural equation modelling. Screening procedures included the assessment of missing data, distributional characteristics, and the identification of potential outliers, following established SEM guidelines (Kline, 2023). Univariate normality was assessed by examining skewness and kurtosis statistics for all observed variables. All values were within recommended thresholds (absolute skewness < 2; absolute kurtosis < 7; Ryu, 2011), indicating that the indicators approximated continuous distributions and that the assumption of approximate normality required for maximum likelihood (ML) estimation in SEM was satisfied. Although the indicators were measured on five- and seven-point Likert-type scales, ML estimation is widely considered acceptable when ordinal variables have five or more response categories and do not exhibit substantial non-normality, as simulation studies indicate that ML provides reliable parameter estimates and model fit statistics comparable to categorical estimation methods (Rhemtulla et al., 2012).

Multivariate outliers were assessed using Mahalanobis distance, with the critical *χ*^2^ value set at *p* < 0.001 (Tabachnick et al., 2007; Xiang et al., 2008); six cases exceeding this threshold were considered multivariate outliers and were excluded to minimize potential distortion of parameter estimates in SEM. After data screening, the final analytical sample consisted of 215 coaches. To assess robustness, the structural model was also estimated using the full sample prior to outlier removal. The pattern of path coefficients and indirect effects was substantively unchanged, suggesting that the main conclusions were not dependent on the exclusion of the six multivariate outliers. Descriptive statistics were computed for all study variables, and Pearson correlation coefficients were calculated to examine initial associations among coach knowledge, psychological safety, coach–athlete relationship quality, and burnout.

### 2.4 Structural equation modelling strategy

A covariance-based structural equation modelling approach was adopted. All analyses were conducted using AMOS version 28.0, following a two-step modelling strategy (Kline, 2023). In the first step, confirmatory factor analysis (CFA) was performed to assess the adequacy of the measurement model. Internal consistency and convergent validity were evaluated using composite reliability (CR) and average variance extracted (AVE), with CR values of 0.70 or higher and AVE values of 0.50 or higher indicating acceptable construct validity (Cheung et al., 2024). Model fit was evaluated using multiple indices, including the chi-square statistic (*χ*^2^), the normed chi-square (*χ*^2^/df), the comparative fit index (CFI), the Tucker–Lewis index (TLI), and the root mean square error of approximation (RMSEA) with 90% confidence intervals. Consistent with established recommendations, CFI and TLI values of 0.90 or above and RMSEA values of 0.08 or below were interpreted as indicative of acceptable model fit (Kline, 2023).

In the second step, the structural model was estimated to test the hypothesized relationships among coach knowledge, psychological safety, coach–athlete relationship quality, and burnout. Standardized path coefficients were examined to evaluate the strength and direction of associations, with effect sizes interpreted in line with conventional SEM guidelines. Because all focal variables were collected via self-report in a single survey administration, a statistical sensitivity analysis was conducted to assess potential common method variance. Following established SEM procedures, a latent common method factor was added to the measurement model, with all indicators loading on both their theoretical construct and the method factor (Richardson et al., 2009).

Also, coaches’ license level was included as an exploratory control variable because coaching qualifications may reflect differences in formal training and professional expertise. In the structural model, license level was specified as a predictor of coach knowledge and burnout.

### 2.5 Mediation analysis

Indirect effects were examined using bias-corrected bootstrap procedures, which provide robust estimates of mediation effects without relying on normality assumptions (Preacher and Hayes, 2008). A total of 5,000 bootstrap resamples were generated to estimate indirect effects and corresponding 95% bias-corrected confidence intervals. Mediation was considered statistically significant when the confidence interval for the indirect effect did not include zero (*α* = 0.05).

Within the structural model, psychological safety was specified as an intermediate mediator linking coach knowledge to coach–athlete relationship quality, which subsequently served as a proximal mediator predicting burnout. This sequential mediation approach enabled a detailed examination of how coach knowledge is translated into burnout outcomes through a combination of contextual appraisals and relational processes.

### 2.6 Common method bias sensitivity analysis

Because all focal variables were collected via self-report in a single survey administration, an additional statistical sensitivity analysis was conducted to assess the potential influence of common method variance. Following common SEM practice, a latent common method factor was added to the measurement model, with all observed items loading on both their theoretical construct and the method factor (Podsakoff et al., 2003; Podsakoff et al., 2012; Richardson et al., 2009). The method factor was specified as orthogonal to the substantive latent constructs. Changes in model fit and in the magnitude of standardized factor loadings and structural paths were examined to evaluate whether the substantive findings were robust to potential shared method variance.

## 3 Results

### 3.1 Descriptive statistics

Descriptive statistics and correlations among CKQ, SPSI, CARTQ, and CBQ are presented in Table 1. CKQ was positively correlated with sport psychological safety (SPSI; *r* = 0.44, *p* < 0.01) and relationship quality (CARTQ, *r* = 0.59, *p* < 0.01). Sport psychological safety was also positively associated with relationship quality (*r* = 0.49, *p* < 0.01). Burnout was negatively correlated with psychological safety (*r* = −0.28, *p* < 0.01) and relationship quality (*r* = −0.18, *p* < 0.05), whereas its correlation with coach knowledge was small and non-significant (*r* = −0.13, *p* = 0.063). These correlations provided preliminary support for the hypothesized relationships and justified subsequent structural equation modelling.

**Table 1:** Means, standard deviations, and correlations among study variables.

Variable	*M*	SD	1	2	3	4
1. CKQ	4.24	0.50	–			
2. SPSI	3.73	0.45	0.44**	–		
3. CARTQ	6.30	0.67	0.59**	0.49**	–	
4. Burnout	2.49	0.68	−0.13	−0.28**	−0.18*	–

M = mean; SD = standard deviation. CKQ = coach knowledge questionnaire; SPSI = sport psychological safety inventory; CARTQ = coach–athlete relationship questionnaire. *p < 0.05. **p < 0.01.

### 3.2 Measurement model

Confirmatory factor analysis (CFA) was conducted to evaluate the adequacy of the measurement model. The model demonstrated an acceptable fit to the data, *χ*^2^(38) = 91.57, *χ*^2^/df = 2.41, CFI = 0.967, TLI = 0.952, and RMSEA = 0.081 (90% CI [0.060, 0.103]; PCLOSE = 0.009; Table 2). Although the RMSEA for the measurement model slightly exceeded the conventional cut-off of 0.08, other incremental fit indices (CFI and TLI) indicated an acceptable model fit. This pattern is consistent with previous SEM research demonstrating that RMSEA may be overly sensitive in complex measurement models with multiple latent constructs, whereas CFI and TLI provide more stable indicators of model fit (Marsh et al., 2004; Kline, 2023). Accordingly, the measurement model was considered acceptable for subsequent structural analysis. All standardized factor loadings were statistically significant (*p* < 0.001), with the majority exceeding 0.70, indicating satisfactory indicator reliability. Convergent validity was assessed by computing the average variance extracted (AVE) for each latent construct. The AVE values indicated acceptable convergent validity for coach knowledge (CKQ = 0.80), psychological safety (SPSI = 0.53), coach–athlete relationship quality (CARTQ = 0.78), and coach burnout (CBQ = 0.67), all exceeding the recommended threshold of 0.50.

**Table 2:** Model fit indices for the measurement and structural models.

Model	*χ* ^2^	df	*χ*^2^/df	RMSEA (90% CI)	PCLOSE	CFI	TLI
Measurement model	91.57	38	2.41	0.081 (0.060–0.103)	0.009	0.967	0.952
Structural model	86.79	38	2.28	0.077 (0.056–0.099)	0.020	0.966	0.951

*χ*^2^ = chi-square; df = degrees of freedom; RMSEA = root mean square error of approximation; CI = confidence interval; PCLOSE = *p* value for test of close fit (RMSEA ≤ 0.05); CFI = comparative fit index; TLI = Tucker–Lewis index.

### 3.3 Structural model and direct effects

The hypothesized structural model demonstrated an acceptable fit to the data, *χ*^2^(38) = 86.79, *χ*^2^/df = 2.28, CFI = 0.966, TLI = 0.951, and RMSEA = 0.077 (90% CI [0.056, 0.099]; PCLOSE = 0.020; Table 2). The inclusion of a latent common method factor did not substantially improve model fit (CFI = 0.965, RMSEA = 0.079) compared with the original measurement model. Moreover, the pattern of factor loadings and structural paths remained largely unchanged. These findings suggest that common method variance is unlikely to fully account for the observed relationships.

To assess the potential influence of multivariate outliers, the structural model was estimated both before and after the exclusion of the six cases identified using Mahalanobis distance. The results obtained from the full sample were largely consistent with those reported in the cleaned dataset. For example, the association between coach knowledge and psychological safety remained positive and significant (*β* = 0.68 vs. *β* = 0.66), and the negative association between relationship quality and burnout was also similar in magnitude (*β* = −0.28 vs. *β* = −0.34). Model fit indices were comparable across the two analyses (full sample: CFI = 0.966, RMSEA = 0.078; cleaned dataset: CFI = 0.965, RMSEA = 0.079). Although some coefficients differed slightly in magnitude, the substantive conclusions of the study were unchanged.

Moreover, to examine alternative directional explanations, an additional model was tested in which burnout was specified as an antecedent rather than an outcome (Burnout → CKQ → SPSI → CARTQ). This alternative model demonstrated identical fit to the hypothesized model (*χ*^2^ = 86.79, df = 38, CFI = 0.966, TLI = 0.951, RMSEA = 0.077), suggesting that the cross-sectional data cannot distinguish between these competing directional interpretations. Nevertheless, the hypothesized model was retained because it aligns more closely with the theoretical framing of coach knowledge as an upstream resource shaping relational and psychological processes within the coaching environment.

As shown in Table 3, coach knowledge was positively associated with psychological safety (*β* = 0.66, *p* < 0.001) and coach–athlete relationship quality (*β* = 0.36, *p* < 0.001). Psychological safety also exhibited a significant positive effect on coach–athlete relationship quality (*β* = 0.45, *p* < 0.001). Coach–athlete relationship quality was negatively associated with burnout (*β* = −0.34, *p* = 0.002). By contrast, the direct effects of psychological safety (*β* = 0.16, *p* = 0.151) and coach knowledge (*β* = −0.02, *p* = 0.860) on burnout were not statistically significant. This pattern indicates that the influence of coach knowledge and psychological safety on burnout may operate primarily through coach–athlete relationship rather than through direct pathways.

**Table 3:** Structural path coefficients of the SEM model.

Path	*B*	SE	*β*	CR	*p*
CKQ → SPSI	0.703	0.080	0.658	8.767	<0.001
CKQ → CARTQ	0.486	0.115	0.358	4.231	<0.001
SPSI → CARTQ	0.568	0.110	0.448	5.172	<0.001
SPSI → Burnout	0.241	0.168	0.163	1.435	0.151
CARTQ → Burnout	−0.401	0.132	−0.344	−3.041	0.002
CKQ → Burnout	−0.029	0.165	−0.018	−0.177	0.860

B = unstandardized coefficient; SE = standard error; *β* = standardized coefficient; CR = critical ratio; Significant paths (*p* < 0.05).

Last, as an exploratory analysis, license level was included as a control variable predicting coach knowledge and burnout. License level showed a small positive association with coach knowledge (*β* = 0.14, *p* = 0.014) but was not significantly related to burnout (*β* = −0.13, *p* = 0.094). Importantly, the inclusion of this control variable did not materially alter the pattern of the main structural relationships.

### 3.4 Indirect effects and mediation analysis

**Figure 1:**
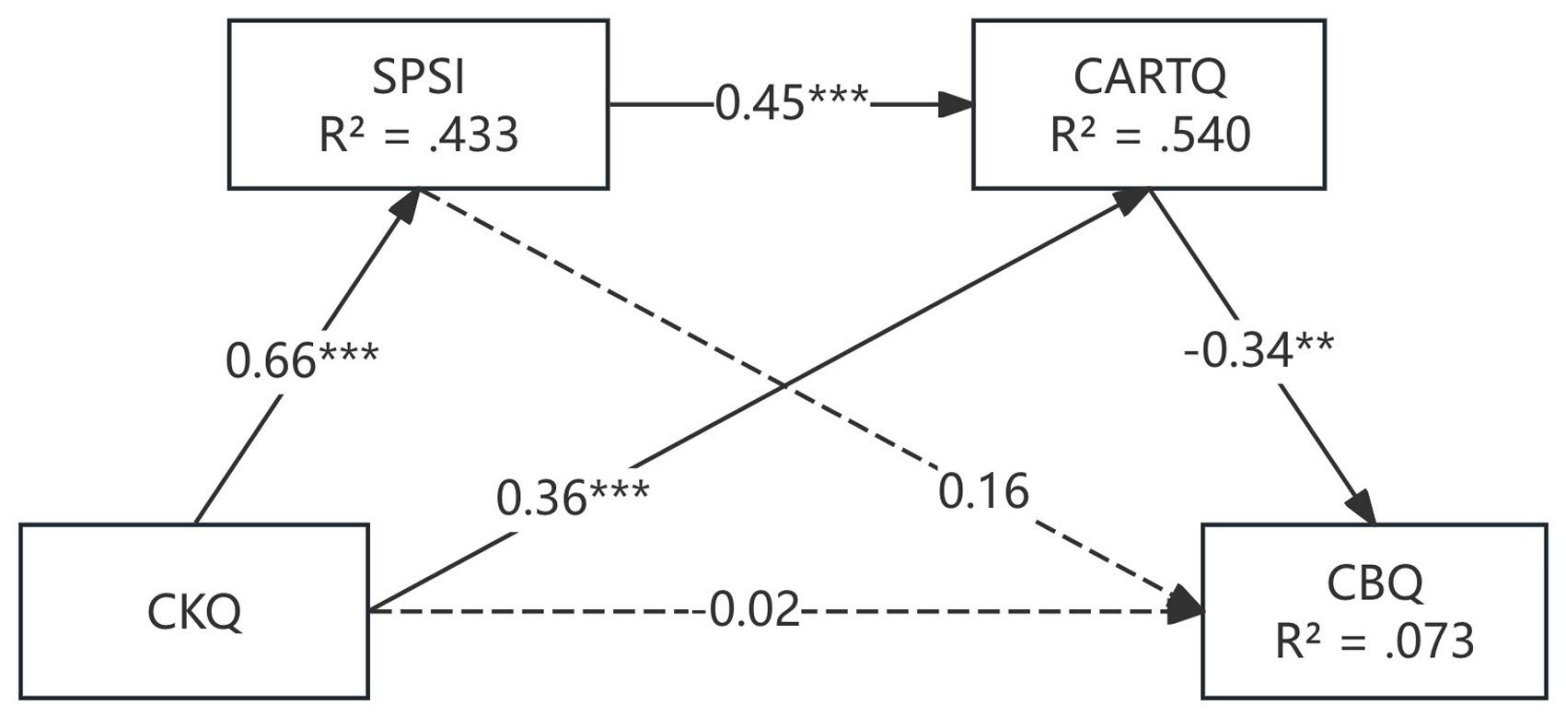
Structural equation model illustrating the hypothesized relationships among coach knowledge (CKQ), psychological safety (SPSI), coach-athlete relationship quality (CARTQ), and burnout. Values represent standardized path coefficients. Solid lines indicate statistically significant paths, whereas dashed lines indicate non-significant paths. Significance levels are denoted as follows: *p* < 0.001, *p < 0.01, *p* < 0.05. *R*^2^ values represent the proportion of variance explained for each endogenous construct.

Indirect effects were examined using bias-corrected bootstrapping with 5,000 resamples (Table 4). Results indicated a significant positive indirect effect of CKQ on CARTQ through SPSI (*β* = 0.400, 95% BC CI [0.234, 0.598]), indicating that higher coach knowledge was associated with greater psychological safety, which in turn related to higher-quality coach–athlete relationships. With respect to burnout, CKQ demonstrated a significant indirect effect through CARTQ alone (*β* = −0.195, 95% BC CI [−0.410, −0.068]), as well as a significant sequential indirect effect through SPSI and CARTQ (*β* = −0.160, 95% BC CI [−0.328, −0.073]). In contrast, the indirect pathway through SPSI alone was not significant (*β* = 0.169, 95% BC CI [−0.048, 0.451]). Overall, the model explained 43.3% of the variance in psychological safety (*R*^2^ = 0.433), 54.0% in coach–athlete relationship quality (*R*^2^ = 0.540), and 7.3% in burnout (*R*^2^ = 0.073). The final structural model with standardized path coefficients is presented in Figure 1.

**Table 4:** Bootstrap results for indirect standardized effects.

Path	Indirect effect	95% BC CI Lower	95% BC CI Upper	*p*
CKQ → SPSI → CARTQ	0.400	0.234	0.598	<0.001
CKQ → SPSI → Burnout	0.169	−0.048	0.451	0.124
CKQ → CARTQ → Burnout	−0.195	−0.410	−0.068	<0.001
CKQ → SPSI → CARTQ → Burnout	−0.160	−0.328	−0.073	<0.001
CKQ → Burnout (direct effect)	−0.029	−0.380	0.306	0.872
CKQ → Burnout (total effect)	−0.186	−0.432	0.074	0.148

BC CI = bias-corrected confidence interval; Significant indirect effects are those with confidence intervals not crossing zero.

## 4 Discussion

### 4.1 Coach knowledge as an antecedent of psychological safety

Consistent with Hypotheses 1, coach knowledge was positively associated with psychological safety. This finding extends dominant conceptualisations of coaching expertise, where knowledge is typically discussed in relation to instructional quality and performance effectiveness, by demonstrating that coach knowledge is also relevant to coaches’ perceptions of the psychological climate in which their work is embedded (Côté and Gilbert, 2009; Lyle and Cushion, 2016). Psychological safety is traditionally defined as individuals’ belief that they can take interpersonal risks—such as speaking up, admitting mistakes, or seeking support—without fear of negative consequences (Edmondson, 1999; Edmondson and Lei, 2014). In elite sport environments, psychological safety extends beyond general interpersonal risk-taking and includes perceptions that the sport setting supports mental health disclosure, promotes mental health literacy, and reduces stigma surrounding psychological difficulties (Rice et al., 2022). Within this perspective, psychological safety reflects whether individuals perceive their sport environment as a mentally healthy and supportive context for recognizing, discussing, and managing mental health challenges.

Psychological safety is shaped by multiple, interacting influences, including organizational expectations and performance pressures, leadership practices, interpersonal relationships, and broader norms regarding disclosure, authority, and error tolerance in elite sport environments (Fransen et al., 2020). Accordingly, even highly knowledgeable coaches may experience reduced psychological safety in contexts characterized by rigid hierarchies, outcome-driven evaluation, or mental health stigma. Nevertheless, the relatively substantial proportion of variance explained in psychological safety in the present model (*R*^2^ = 0.433) indicates that coach knowledge represents a meaningful, though not exclusive, contributor to coaches’ perceptions of psychological safety in high-level football environments.

In line with coaching expertise frameworks, knowledge resources may therefore contribute to psychological safety by enhancing coaches’ capacity to contextualize challenges and engage more confidently with support systems, rather than by directly eliminating stressors (Côté and Gilbert, 2009; Lyle and Cushion, 2016). Coaches with more developed professional expertise, interpersonal competence, and reflective capacity are likely to possess stronger sense-making abilities, enabling them to contextualize performance pressures, evaluative demands, and interpersonal challenges as manageable features of the coaching role rather than as unpredictable threats. This cognitive capacity may facilitate more confident engagement with organizational and interpersonal support systems (Rice et al., 2022). Therefore, coach knowledge may function as an upstream resource that enables coaches to recognise psychological risks, understand available support pathways, and engage more constructively with psychologically informed practices. These capacities may contribute to stronger perceptions of a mentally healthy environment, greater mental health awareness, and lower self-stigma within the coaching context. Accordingly, coach knowledge may enhance perceptions of psychological safety not only by strengthening interpersonal confidence, but also by supporting the development of psychologically supportive norms and practices within elite sport environments. From an applied perspective, these findings suggest that coach education should extend beyond technical instruction to include reflective practice, decision rationales, and psychologically informed coaching that supports mentally healthy, literacy-enhancing, and low-stigma sport settings.

### 4.2 Psychological safety as a pathway linking coach knowledge to coach–athlete relationship quality

Consistent with Hypotheses 2, psychological safety partially mediated the association between coach knowledge and coach–athlete relationship quality. Pressures in the elite sport environments may shape how coach knowledge is enacted within interpersonal relationships, rather than determining relational quality in isolation. In the present model, coach knowledge was positively associated with coach–athlete relationship quality both directly and indirectly via psychological safety, indicating that knowledge resources contribute to relational functioning through multiple, complementary pathways. The relatively strong proportion of explained variance in relationship quality (*R*^2^ = 0.540) underscores the importance of psychological safety as a meaningful contextual mechanism through which coach knowledge is relationally enacted, while also coexisting with a direct knowledge–relationship link. This pattern aligns with relational models of coaching, which emphasize that effective relationships are shaped both by coaches’ personal resources and by the psychological conditions under which these resources are expressed (Jowett, 2017; Jowett et al., 2023).

The significant direct association between coach knowledge and relationship quality further suggests that professional expertise and reflective capacity are not merely technical assets, but also relational resources. Coaches with more developed knowledge bases may be better equipped to interpret performance expectations, justify coaching decisions, and navigate the inherent uncertainties of elite sport contexts, thereby supporting clearer role expectations, greater relational stability, and mutual confidence in the coaching partnership (Côté and Gilbert, 2009; Gilbert and Côté, 2013). From this perspective, coach knowledge facilitates relationship quality by enhancing credibility, decision confidence, and shared understanding, core relational elements underpinning closeness, commitment, and coordinated collaboration in the coach–athlete relationship (Jowett and Poczwardowski, 2007; Rhind and Jowett, 2012).

At the same time, coach knowledge may further influence relationship quality by shaping coaches’ perceptions of psychological safety. Greater professional knowledge and reflective capacity may support higher levels of perceived psychological safety, particularly in domains related to mental health literacy and perceptions of a mentally healthy environment (Rice et al., 2022). Recent evidence underscores that psychological safety is a robust predictor of interpersonal relationship quality (Edmondson and Lei, 2014; Jowett et al., 2023). For example, Jowett et al. (2023) demonstrated that when athletes perceive their team or subgroup environment as psychologically safe, they are more likely to experience trusting, respectful, and cooperative relationships with their coaches. Extending this logic, coaches’ own perceptions of psychological safety may play a parallel enabling role in the formation of high-quality coach–athlete relationships, that support effective collaboration under performance demands (Hampson and Jowett, 2014; Jowett, 2017; Jowett and Poczwardowski, 2007). In this sense, psychological safety may be understood as what Edmondson (2018) describes as an “engine” rather than a “fuel” for performance—not a direct driver of outcomes, but a foundational condition that enables effective coaching processes to operate.

### 4.3 Coach-athlete relationship quality as a mediating mechanism linking coach knowledge and coach burnout

Consistent with Hypothesis 3, coach-athlete relationship quality mediated the association between coach knowledge and coach burnout, indicating that knowledge resources influence burnout primarily through their implications for relational functioning rather than through a direct pathway. This finding aligns with relational perspectives in coaching research, which position the coach–athlete relationship as a central process through which broader psychological and contextual factors are translated into wellbeing outcomes (Nicholls and Perry, 2016; McShan and Moore, 2023). High quality coach-athlete relationships represent an important relational resource for coaches operating in high-performance environments. Jowett (2017) positions the coach–athlete relationship as the mechanism through which coach knowledge is enacted in practice. Knowledge informs planning, decision-making, and intentions, but it is the relationship that enables this knowledge to be expressed through everyday coaching work, including guidance, communication, coordination, and role alignment. From the coach’s perspective, such relationships may buffer against chronic emotional strain by providing relational stability, mutual trust, and a sense of shared purpose with athletes (Isoard-Gautheur et al., 2016). These relational features represent precisely the interpersonal conditions that can reduce chronic relational friction and emotional labor among both coaches and athletes (Lafrenière et al., 2011; Olusoga et al., 2019; McShan and Moore, 2023; Pacewicz et al., 2023; Şenel et al., 2025).

The mediating role of coach–athlete relationship quality is best understood as an explanatory process through which coach knowledge becomes consequential for coach burnout. Within the coaching literature, knowledge has been consistently linked to core resources that shape coaches’ effectiveness, professional competence, and capacity to manage sustained performance demands (Côté and Gilbert, 2009; Gilbert and Côté, 2013). Recent work further conceptualizes coach knowledge as a form of role proficiency (Stewart et al., 2024a). Central to this proficiency is not only technical expertise, but also the ability to engage in effective and flexible interpersonal interaction within dynamic performance environments, a feature long emphasized in coaching effectiveness frameworks (Côté and Gilbert, 2009). Empirical evidence indicates that high-quality relationships facilitate knowledge sharing, the development of shared understanding, and coordinated goal pursuit, all of which are critical for effective performance functioning (Stewart et al., 2024a; Stewart et al., 2024b). From this perspective, coaches with stronger knowledge resources may be better positioned to establish and sustain functional relationships with athletes, as knowledge supports credibility, role clarity, and confidence in relational engagement. These relational processes are particularly salient when considering coach burnout, reflecting the cumulative erosion of perceived effectiveness and engagement in the coaching role (Raedeke and Smith, 2001). When coach–athlete relational functioning is stronger, coaches may be more likely to experience their work as meaningful, effective, and aligned with shared performance goals, thereby buffering against professional burnout. Accordingly, the present findings suggest that coach–athlete relationship quality functions as a relational conduit linking coach knowledge to burnout outcomes. Rather than acting independently, knowledge resources appear to be associated with lower burnout primarily through relational processes that sustain coaching effectiveness over time.

### 4.4 Sequential mediation of psychological safety and coach–athlete relationship quality

Hypothesis 4 proposed that the association between coach knowledge and coach burnout would be explained through a sequential mediation process involving psychological safety and coach–athlete relationship quality. The findings provided support for this hypothesis. This pattern suggests that psychological safety may reflect an important cognitive–evaluative appraisal of one’s working environment, but is not sufficient, in isolation, to alter coaches’ longer-term burnout trajectories. In the present sample, elite football coaches generally reported high levels of knowledge, which appeared to shape how they appraised the psychological climate of their working environment under performance pressure. However, such appraisals, reflected in perceived psychological safety, did not on their own translate into reduced burnout.

A significant indirect effect emerged only when psychological safety was followed by coach–athlete relationship quality, highlighting the importance of a relational pathway. Coach knowledge may help coaches manage the demands of performance outcomes and interpersonal expectations. From this perspective, coach knowledge can be understood as a latent resource, whose influence on wellbeing is not automatic but depends on contextual and relational activation within the coaching environment. Psychological safety functions as a necessary intermediate layer through which coaches’ knowledge becomes experientially meaningful, shaping how interpersonal demands, performance pressure, and evaluative scrutiny are appraised in elite sport environments (Rice et al., 2022; Vella et al., 2024).

Coach–athlete relationship quality captures trust, respect, relational investment, and goal-oriented relational commitment (Jowett and Poczwardowski, 2007). While psychological safety may enable constructive interaction by shaping whether the environment is perceived as safe enough for coordination and communication, relationship quality determines whether such interactions are sustained, emotionally viable, and meaningful under chronic performance pressure. In this sense, psychological safety reflects the perceived conditions for interaction, whereas relationship quality captures the relational realization of these conditions within the coach’s most central working partnership. When this relational conversion occurs, coaches are more likely to experience their role as coherent, supported, and efficacious, thereby reducing the likelihood of the exhaustion, devaluation, and reduced accomplishment that define burnout. This interpretation aligns with broader burnout research. Occupational burnout is commonly addressed through two complementary pathways: designing more humane and supportive work environments, and enhancing individuals’ capacity to tolerate or regulate person–job mismatches (Maslach and Leiter, 2016).

In high-performance sport, high-quality coach–athlete relationships can be understood as a key manifestation of a more humanized coaching workplace. Specifically, burnout is increasingly conceptualized as emerging from sustained transactions between individuals and their social environments, suggesting that contextual resources such as supportive climates must be translated into day-to-day relational functioning to exert protective effects (Gustafsson et al., 2017; Leiter, 2018; Schaufeli, 2006). Such relationships not only provide emotional support and interpersonal affirmation, but also enhance coaches’ perceived sense of control through clear role expectations, stable interaction patterns, and goal-oriented relational commitment. Within the areas-of-worklife framework, perceived control is a central mechanism through which work–person mismatches are reduced, thereby lowering vulnerability to burnout (Leiter and Maslach, 2016). Accordingly, strong coach–athlete relationships may buffer burnout not merely by improving relational experiences per se, but by increasing coaches’ perceived control over their work environment, which mitigates the cumulative psychological strain associated with sustained performance demands.

Importantly, the modest proportion of variance explained in burnout (*R*^2^ = 0.073) suggests that the present model captures only a limited aspect of the broader processes contributing to coach burnout. While the model identifies a theoretically coherent relational pathway linking coach knowledge, psychological safety, coach–athlete relationship quality, and burnout, this pathway should be understood as one component within a more complex and multifactorial burnout process. Contemporary burnout research consistently conceptualizes burnout as a multifactorial phenomenon shaped by the interaction of personal, relational, and organizational conditions (Gustafsson et al., 2017; Leiter, 2018; Schaufeli, 2006). Coaches are typically exposed to sustained performance pressure, long working hours, and emotionally demanding interpersonal responsibilities, all of which contribute to chronic strain over time (Fletcher and Scott, 2010). Burnout reflects the cumulative impact of multiple organizational and occupational demands within high-performance sport environments (Olusoga et al., 2019; Raedeke and Kenttä, 2013). Excessive workload and insufficient recovery opportunities have been consistently associated with emotional exhaustion among high-performance coaches (Bentzen et al., 2016), while role conflict and role ambiguity represent additional occupational stressors that may further contribute to burnout symptoms in coaching contexts (Fletcher and Scott, 2010). These findings therefore suggest that coach knowledge may represent a distal or contextual resource rather than a primary determinant of burnout.

Accordingly, the present findings should be interpreted as identifying one relational mechanism within a broader system of determinants influencing coach burnout. While psychological safety and coach–athlete relationship quality may represent important interpersonal processes shaping coaches’ experiences of their work environment, structural and organizational conditions are likely to play an equally important role. Future research integrating relational variables with organizational factors such as workload, recovery opportunities, and perceived autonomy may provide a more comprehensive understanding of burnout processes among high-performance coaches.

## 5 Conclusion

This study examined how coach knowledge relates to coach burnout through a sequential mediation model involving psychological safety and coach–athlete relationship quality. The findings demonstrate that coach knowledge is associated with burnout not through direct effects, nor through psychological safety alone, but through a relational pathway in which psychological safety facilitates knowledge to be enacted in high-quality coach–athlete relationships. This highlights the importance of distinguishing between cognitive resources, contextual appraisals, and relational functioning when explaining coach wellbeing. Conceptually, the findings extend coaching effectiveness and burnout models by demonstrating that coach knowledge contributes to wellbeing through relational pathways, and that psychological safety further strengthens this process by shaping the conditions under which knowledge is enacted within coach–athlete relationships. Practically, the findings suggest that interventions aimed at reducing coach burnout should move beyond knowledge development alone, and instead focus on fostering psychologically safe climates and sustaining high-quality coach–athlete relationships. By identifying relationship quality as a key proximal mechanism, this study contributes to a more process-oriented understanding of coach burnout in high-performance sport.

### 5.1 Limitations and future directions

Several limitations should be acknowledged. First, the present study relied on a cross-sectional design, which restricts strong conclusions regarding temporal or causal relationships between variables. Although the proposed model was theoretically grounded, the equivalent fit of the alternative model highlights that burnout may also shape coaches’ perceptions of their work environment and relational experiences. Future longitudinal or time-lagged research would be valuable for clarifying the dynamic relationships between coach knowledge, relational processes, psychological safety, and burnout in coaching environments. Second, all variables were assessed via self-report at a single time point. A latent common method factor analysis indicated that including a method factor did not materially change the model estimates, suggesting that common method variance was unlikely to drive the observed relationships. Future studies could further address this issue using multi-source data. Third, the sample focused on high-level football coaches, which may limit generalisability to other sports or performance levels. Future studies should test this model across diverse coaching contexts and explore additional organizational and recovery-related factors that may interact with relational mechanisms. Integrating qualitative approaches may also provide deeper insight into how coaches translate knowledge into relational practice under sustained performance pressure.

## Data Availability

The study was reviewed and approved by the Ethics Committee of Shanghai University of Sport (Approval No. 102772025RT313). Written informed consent was obtained from all participants prior to their participation in the study.
